# E-Cadherin and FGFR1 Expression in Mouse Osteoblastogenesis in Normoxic Cultures

**Published:** 2017-03

**Authors:** Osama Al-Amer

**Affiliations:** Department of Medical Laboratory Technology, Faculty of Applied Medical Sciences, University of Tabuk, Tabuk, Saudi Arabia

**Keywords:** Osteoblast differentiation, E-cadherin and FGFR1

## Abstract

E-cadherin is a cell surface adhesion molecules that play an important role in tissue differentiation. FGFR1 is expressed in the developing and mature skeleton in patterns suggestive of both unique and redundant function. Expression levels of E-cadherin and FGFR1 during osteoblastogenesis unclear. In this study primary calvarial mouse osteoblasts were differentiated to mature osteoblasts in osteogenic medium. Alkaline phosphatase (ALP) activity, alizarin red staining, gene expression (Runt-related transcription factor 2 (Runx2), collagen 1 (COL1A2), osteocalcin, E-cadherin and FGFR1) and protein expression (E-cadherin and FGFR1) of osteogenic-cultured primary mouse osteoblast were analysed in this study. The osteogenesis capacity of primary osteoblasts was significantly promoted as ALP activity, alizarin red staining, and the relative expression of Runx2 mRNA and COL1A2 mRNA significantly increased during osteoblastogenesis. The results demonstrated that E-cadherin mRNA and protein were expressed in immature osteoblasts (day 7), but not in mature osteoblasts (day 28). In contrast, the expression of FGFR1 mRNA and protein significantly highly expressed in mature osteoblasts (day 28) compared with immature osteoblasts (day 7). In conclusion, this study demonstrated that E-cadherin could be used as a marker for immature osteoblasts, whereas FGFR1 could be used as a marker for mature osteoblasts during *in vitro* osteoblastogenesis.

## INTRODUCTION

Osteoblasts are mononucleated cells derived from putative mesenchymal stem cells (MSCs) in the bone marrow, which are responsible for bone formation. Their functions include regulated synthesis and mineralization of the bone matrix as well as bone remodeling through the activation of osteoclasts. The differentiation pathway is further characterized by sequential expression of specific genes that code for collagenous and noncollagenous bone proteins, cytokines, cytokine receptors, and hormone receptors. Bone morphogenic proteins (BMPs), Wnt/β-catenin and Notch signalling all play a major role in osteoblastogenesis (1-3). In addition, Runx2 has been shown to be an essential transcription factor for osteoblastogenesis (4). Mature osteoblasts are identified by their cuboidal structure and their location on the endosteal surface. Type I collagen is the major product of the bone-forming osteoblast (5).

Epithelial (E-) cadherin play an essential role in Ca^2+^-dependent cell-to-cell adhesion. A striking feature of the type I (classic) cadherins is a highly conserved His-Ala-Val (HAV) motif in the first extracellular domain that mediates homophilic interactions between cadherin molecules expressed on adjacent cells (6). On the other side of the cell membrane, the cadherin intracellular domain forms a protein complex with catenin molecules, which anchor the cadherins to the cytoskeleton and provide a unique signal transduction pathway for cadherin interactions (7). A limited repertoire of E-cadherin has been identified in osteoblastogenesis.

Fibroblast growth factor receptors (FGFRs) play major roles in skeletogenesis, and activating mutations of the human *FGFR1, FGFR2* and *FGFR3* genes cause premature fusion of the skull bones (craniosynostosis) (8). This genetic evidence establishes a role for FGFR1 in skeletal development and suggests that FGFR1, FGFR2 and FGFR3 signaling pathways may have similar or redundant functions. In addition to premature fusion of cranial sutures, several of the classic craniosynostosis syndromes have associated phenotypes that affect long bone development in the appendicular skeleton (9). FGFRS 1–3 are expressed in the developing and mature skeleton in patterns suggestive of both unique and redundant function (10). In the developing growth plate, both FGFR1 and FGFR2 are expressed in condensing mesenchyme that will give rise to cartilage. FGFR2 remains expressed in reserve chondrocytes and appears to be down regulated in proliferating chondrocytes, whereas FGFR1 is expressed in hypertrophic chondrocytes. Later in development, FGFR1 and FGFR2 are both expressed to give rise to osteoblasts and cortical bone. In contrast to FGFR1 and FGFR2, FGFR3 is prominently expressed in proliferating chondrocytes where it regulates cell growth and differentiation and in differentiated osteoblasts where it regulates bone density and cortical thickness. Moreover, FGFR1 found to be necessary in osteoblasts to maintain the balance between bone formation and remodeling through a direct effect on osteoblast maturation (11, 12).

In this study, mouse primary osteoblasts were extracted from mice skull and differentiated into mature osteoblasts. Then, the expression levels of E-cadherin and FGFR1 were examined at different stages of osteoblastogenesis. This study showed FGFR1 mRNA was expressed in primary osteoblasts at different stages of differentiation; days 7, 14, 21 and 28, and demonstrated a positive correlation of increased FGFR1 mRNA expression during osteoblastogenesis. On the other hand, this study showed that E-cadherin only expressed in immature osteoblasts but not in mature osteoblasts.

## MATERIALS AND METHODS

### Mouse primary osteoblast preparation and culture

Osteoblasts were extracted as in previous standard published methods (13, 14). Briefly, calvarial bones were obtained from 2-4 day old C57Bl/6J mice to extract mouse primary osteoblasts. Calvariae were dissected into small pieces and then digested with trypsin-ethylene-diaminetetraacetic acid (EDTA) for 10 minutes at 37°C with shaking then centrifuged at 1100 rpm for 1 min after which the supernatant was discarded. Tissues were incubated with 0.5% of 280 units/mg collagenase II in PBS, 0.15 g of collagenase II was added into 30 ml of PBS containing 100 units/ml Penicillin / 100 μg/ml of Streptomycin and 1.36 μg/mL of amphotericin B, for 10 minutes then centrifuged at 1100 rpm for 1 min after which the supernatant was discarded. Tissues were incubated with 0.5% of 280 units/mg collagenase II in PBS for 10 minutes then centrifuged at 1100 rpm for 1 min after which the supernatant was collected. This step was repeated 4 times and then the cells were pooled and re-suspended in MEM alpha containing 10% FCS, 100 units/ml Penicillin / 100 μg/ml of Streptomycin and 1.36 μg/mL of amphotericin B. After 6 hours, osteoblasts adhered to the bottom of the flasks while other cells floated in the medium. All procedures were carried out under personal license (40/10118).

### Differentiation of osteoblasts

**Figure 1:**
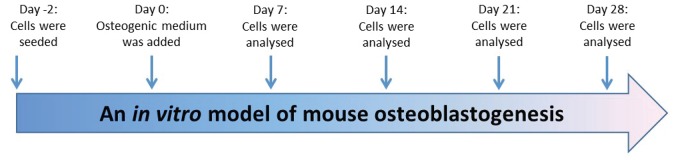
Experiment design of mouse osteoblastogenesis. Extracted osteoblasts from mouse calvariae were seeded in T-75 flasks for expansion of cell numbers. Day -2; 6 × 10^3^ cells/cm^2^ of mouse primary osteoblasts was re-suspended in 10% α-MEM medium and seeded in 96 well plates, 24 well plates and flasks. Cells were then incubated at 37°C with 5% CO_2_. Osteoblasts were differentiated using osteogenic medium. Day 0; α-MEM medium was replaced by osteogenic medium to start osteoblastogenesis process. Osteoblastogenesis was analyzed at days 7, 14, 21 and 28 using ALP activity, alizarin red staining, RT-PCR, TaqMan, and western blot experiments. Medium was changed every 2 days.

Primary osteoblasts were differentiated for 4 weeks from immature osteoblasts to mature osteoblasts in osteogenic media; MEM alpha containing 10% FCS, 10 mM of β-glycerophosphate, 50 μg/ml of ascorbic acid as described previously (Figure 1) (13, 15, 16). Alkaline phosphatase (ALP) activity was used as an osteoblast differentiation marker and alizarin red staining was used as a maturation marker, in which alizarin red dye binds to calcium in matrix deposited by mature osteoblasts.

### Alkaline phosphatase (AKP) activity detection

p-Nitrophenyl phosphate (pNPP) (Sigma, UK) is a soluble substrate used for the detection of alkaline phosphatase activity. 6×10^3^ cells/cm^2^ of primary osteoblasts were seeded in 96 well plates in MEM alpha containing 10% FCS, 100 units/ml Penicillin / 100 μg/ml of Streptomycin and 1.36 μg/mL of amphotericin B. Cells were washed twice with ice cold phosphate buffered saline (PBS) and permeabilised with 20 μl of 0.1% triton with agitating for 20 minutes at room temperature. ALP activity was determined by reading the absorbance at A405 every 5 minutes for 90 minutes and using the following formula; then, ALP activity was normalized with osteoblast DNA concentration using PicoGreen® double-stranded DNA quantitation reagent (Invitrogen, UK).

$$\text{ALP}\;\text{activity}=\frac{({\text{OD}}_{\text{(t1)}}-{\text{OD}}_{\text{(t0)}})\times 1000\times \text{Total}\;\text{volume}}{\text{Time}\times \text{ε}(18.75)\times \text{Path}\;\text{length}(0.639)\times \text{Sample}\;\text{volume}}$$

OD_(t1)_, last optical density; OD_(t0)_, start optical density; ε, constant; path length, length of 200 volume of sample in 96 well plate.

### Alizarin red staining assay

Alizarin red staining is a dye used as an *in vitro* mineralisation marker, in which alizarin red dye binds to calcium in matrix deposited by osteoblasts. 6 × 10^3^ cells/cm^2^ of primary mouse osteoblasts were seeded in 24 well plates in MEM alpha containing 10% FCS, 100 units/ml penicillin / 100 μg/ml of streptomycin and 1.36 μg/mL of amphotericin B. Cells were washed twice with ice cold PBS and fixed with 1 ml of ice cold 70% ethanol for 1 hour at 4°C. Cells were rehydrated with water for 5 minutes before 1 ml of 1% alizarin red (pH 4.2) was added to each well for 10 minutes, washed 5 times with water and dried overnight to at 25°C. ImageJ software was used to quantify the red colour of alizarin red staining. Colour images were converted into 8 bit images (255 grey levels) and the intensity of objects and pixel value of white/black was calculated.

### RT-PCR

Reverse transcriptase polymerase chain reaction was used to assess FGFR1 and E-cadherin mRNA expression. Total RNA was isolated from primary osteoblasts at different stages of development using TriZol. RNAs were transcribed to cDNA using SuperScript^TM^ II reverse transcriptase (Invitrogen, UK). RT-PCR amplifications used the following set of primers:

Gapdh: F-5′-TTGTCAGCAATGCATCCTGC-3′   R-5′-GCTTCACCACCTTCTTGATG-3′

FGFR1: F-5′-CACATCGAGGTGAACGGGAGTAAG-3′   R-5′-CGCATCCTCAAAGGAGACATTCC-3′

E-cadherin: F-5′-TGGAGGAATTCTTGCTTTGC-3′   R-5′-CGTACATGTCAGCCAGCTTC-3′

### TaqMan analysis

Quantification of COL1A2, Runx2, osteocalcin, FGFR1 and E-cadherin mRNA were performed using TaqMan probes for COL1A2, Runx2, osteocalcin, FGFR and E-cadherin, respectively, with TaqMan Universal master Mix II (Applied biosystems, UK). ABI PRISM > 900 sequence detector was used to quantify the expression of COL1A2, Runx2, osteocalcin, FGFR1 and E-cadherin during primary osteoblasts differentiation.

### Western blot

Proteins were extracted from ~80% confluent cells and quantified using the BCA protein assay. 10 μg of protein samples were separated on 12.5% SDS-PAGE, blotted on PVDF membranes and probed with anti-FGFR1 antibody ab76464 (Abcam, UK) at 1:1000 dilution, anti-E-cadherin antibody ab15148 (Abcam, UK) 1:1000 dilution or with anti-β-actin antibody ab8227 (Abcam, UK) as housekeeping genes. Anti-rabbit IgG antibodies horseradish peroxidase-linked species-specific (GE Healthcare, UK) at 1:2000 dilution was used as secondary antibody.

### Statistical analysis

Statistical significance was determined using non-parametric Kruskall-Wallis, analysed using GraphPad Prism 6 Software. Data was considered significant if *p*=<0.05 (*p<0.05, **p<0.01, ***p<0.001). All data were presented using the standard diviasion (± SD).

## RESULTS

### Development a model of mouse osteoblastogenesis

**Figure 2:**
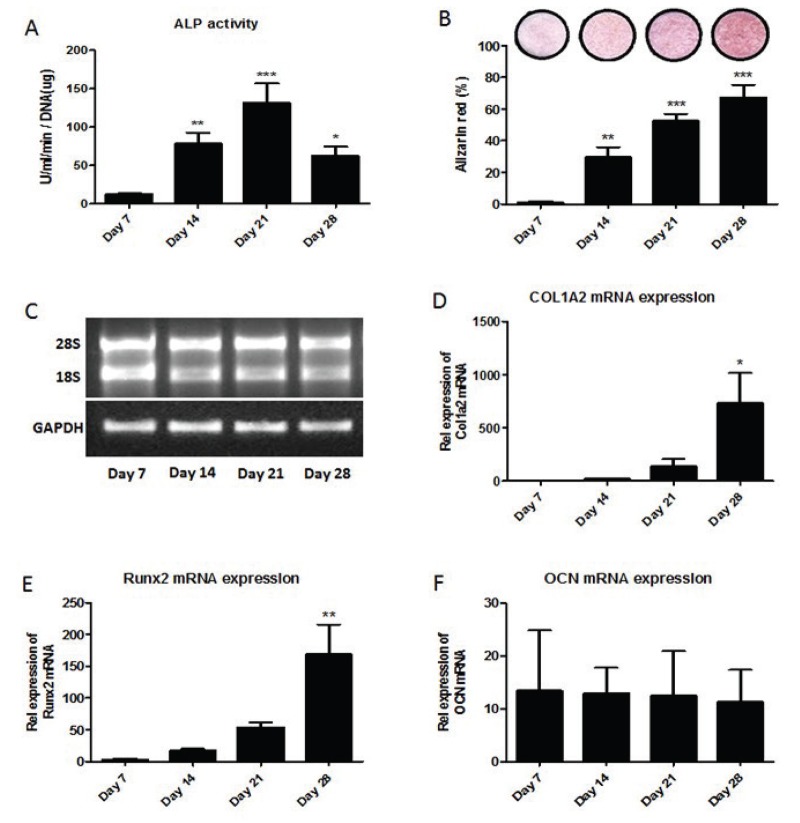
Mouse primary osteoblastogenesis. Primary osteoblasts were differentiated over 4 weeks in osteogenic medium. Panel A shows significant increases in the ALP activity, an osteoblast differentiation marker, during osteoblastogenesis on day 14 and 21. Panel B shows significant increases in the alizarin red staining, an osteoblasts maturation marker, during osteoblastogenesis. Panel C shows electrophoresis of total mRNA isolated from cells and RT-PCR of GAPDH mRNA during osteoblastogenesis. The relative expression of osteoblast marker genes; COL1A2 (panel D), Runx2 (panel E) and osteocalcin (OCN) (panel F) expression during osteoblastogenesis were analyzed using TaqMan.

Differentiation of mouse primary osteoblasts was analyzed using alkaline phosphatase activity (ALP), alizarin red staining, and the level of expression of osteoblast markers; Runx2, COL1A2 and osteocalcin genes. Cells were seeded and primary osteoblasts were analyzed at day 7 (proliferation), day 14 (differentiation), day 21 (maturation) and day 28 (mineralization). Figure 2A shows statistically significant increases in ALP activity of primary osteoblast on day 14 and on day 21 compared to day 7. On day 28, ALP activity was significantly decreased compared to ALP activity on day 21. Figure 2B shows alizarin red staining statistically significant increased during osteoblastogenesis as osteoblasts become fully mature. Total RNA was extracted over 4 weeks differentiation of primary osteoblasts cultured *in vitro*. Figure 2C shows the electrophoresis of RNA isolated from primary osteoblasts during differentiation; days 7, 14, 21 and 28, and RT-PCR of GAPDH in differentiated mouse primary osteoblasts after reverse transcription. The level of expression of osteoblast markers; COL1A2 (Figure 2D), Runx2 (Figure 2E) and osteocalcin (Figure 2F) genes were analyzed using TaqMan. Data showed statistically significant increases in the relative expression of Runx2 mRNA and Runx2 mRNA on day 28 compared to day 7. However, there was no difference in the relative expression of Osteocalcin mRNA during osteoblastogenesis.

### FGFR1 and E-cadherin expression during osteoblastogenesis

**Figure 3:**
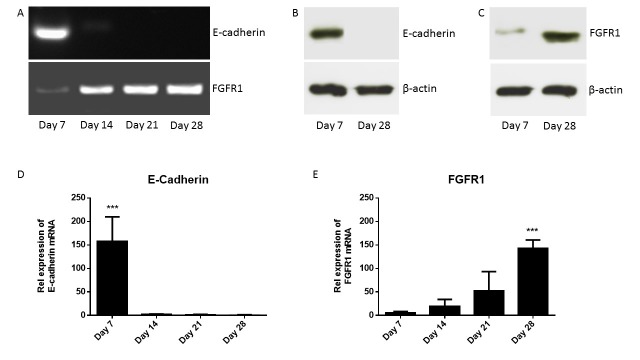
Expression of E-cadherin and FGFR1 during osteoblastogenesis. Primary osteoblasts were differentiated over 4 weeks in osteogenic medium. Panel A shows the expression of E-cadherin and FGFR1 mRNA during osteoblastogenesis. Panel B shows the expression of E-cadherin protein in immature (day 7) and mature (day 28) osteoblasts using western blotting. Panel C shows the expression of FGFR1 protein in immature (day 7) and mature (day 28) osteoblasts using western blotting. Panel D shows the relative expression of E-cadherin during osteoblastogenesis using TaqMan. Panel E shows the relative expression of FGFR1 during osteoblastogenesis using TaqMan.

cDNAs were synthesized from mouse primary osteoblasts cultured *in vitro* for over 4 weeks. Figure 3A shows the expression of FGFR1 and E-cadherin during osteoblastogenesis. Data showed that E-cadherin mRNA was expressed in primary osteoblasts only in early stage of differentiation, immature osteoblasts (day 7), while FGFR1 mRNA was expressed in primary osteoblasts at different stages of differentiation; days 7, 14, 21 and 28. Figure 3B shows that E-cadherin protein was expressed in immature osteoblasts (day 7) but not in mature osteoblasts (day 28) using western blotting. Figure 3C shows that FGFR1 protein was expressed in immature osteoblasts (day 7) and in mature osteoblasts (day 28) using western blot. However, data showed that there is an increase in the relative expression of FGFR1 protein in mature osteoblasts (day 28) compared with immature osteoblasts (day 7) using western blotting.

The quantitation expression of E-cadherin and FGFR1 mRNA was analyzed using TaqMan analysis. Expression of E-cadherin and FGFR1 was normalized to expression of the housekeeping gene, β-actin. Figure 3D shows the relative expression of E-cadherin mRNA during primary osteoblasts differentiation. Data shows significant increase in the relative expression of E-cadherin mRNA in immature osteoblasts (day 7) compared with mature osteoblasts (day 28). Figure 3E shows the relative expression of FGFR1 mRNA during primary osteoblasts differentiation. Data shows that a positive correlation of increased FGFR1 mRNA expression during osteoblast differentiation over 4 week. Moreover, data shows significant increase in the relative expression of FGFR1 mRNA in mature osteoblasts (day 28) compared with immature osteoblasts (day 7). These data suggested that E-cadherin could be used as a marker for immature osteoblasts while FGFR1 could be used as a marker for mature osteoblasts.

## DISCUSSION

In this study an *in vitro* model of mouse osteoblastogenesis was developed. Primary cultures of calvarial mouse osteoblasts were differentiated to mature functional osteoblasts as previous published method. ALP activity and alizarin red staining were used as an osteoblast differentiation and mineralization markers. Xiao *et al* (2006) demonstrated that ALP activity and alizarin red staining increased during mouse osteoblastogenesis (16). In addition, Hay *et al* also showed that ALP activity and alizarin red staining increased during mouse osteoblastogenesis (14). In this study, ALP activity and alizarin red staining were also used as osteoblast differentiation and mineralization markers. My experiments also showed the ALP activity and alizarin red staining increased during mouse osteoblastogenesis.

The quantification of relative levels of expression of Runx2 mRNA, COL1A2 mRNA and osteocalcin mRNA was analyzed to determine the relevance between osteoblast markers, Runx2 collagen I (COL1A2), and osteocalcin during osteoblastogenesis. Runx2 is a transcriptional activator necessary for bone formation and osteoblast differentiation in mice (17). Runx2-null mice resulted in a complete lack of bone formation and suggested that Runx2 plays an essential role in osteoblastogenesis in mice (18). My experiments demonstrated that the relative expression of Runx2 mRNA significantly increased during osteoblastogenesis. COL1A2 gene encodes one of the chains for type I collagen and mutations in this gene produced an osteogenesis imperfecta a type IV phenotype (19). Both Runx2 and COL1A2 are induced at an early stage in osteoblast differentiation (20). My experiments demonstrated that the relative expression of COL1A2 mRNA significantly increased during osteoblastogenesis. Therefore, this study showed that not only ALP activity and alizarin red staining are useful as osteoblast differentiation and mineralization markers, but also the relative expression of Runx2 mRNA and COL1A2 mRNA can be used as markers for mouse osteoblastogenesis.

E-cadherin play an essential role in Ca^2+^-dependent cell-to-cell adhesion and a limited repertoire of E-cadherins has been identified in osteoblastogenesis. FGFR1 is expressed in the developing and mature skeleton in patterns suggestive of both unique and redundant function, however, their expression level during osteoblastogenesis unclear. In this study specific primers and an antibody against E-cadherin and FGFR1 were used to analyze the relative expression of E-cadherin and FGFR1 mRNA and protein, respectively, during osteoblastogenesis. TaqMan analysis of primary calvarial osteoblasts cDNA demonstrated highly expression of E-cadherin mRNA in immature osteoblasts (day 7) only. The data showed a significant increase in the E-cadherin mRNA expression in immature osteoblasts (day 7) compared with mature osteoblasts (day 28), which is completely negative. In addition, TaqMan analysis of primary calvarial osteoblasts cDNA demonstrated an increase in the expression of FGFR1 mRNA during osteoblastogenesis from day 7 to day 28. The data showed a significant increase in the FGFR1 mRNA expression in mature osteoblasts (day 28) compared with immature osteoblasts (day 7), which is very low.

## CONCLUSION

These results showed a high levels of E-cadherin expression in immature osteoblasts and relatively no expression of E-cadherin in mature osteoblasts during osteoblastogenesis. In contrast, the results showed a very low levels of FGFR1 expression in immature osteoblasts and relatively high levels of FGFR1 expression in mature osteoblasts during osteoblastogenesis. In conclusion, this study demonstrated that E-cadherin could be used as a marker for immature osteoblasts during *in vitro* osteoblastogenesis, whereas FGFR1 could be used as a marker for mature osteoblasts during *in vitro* osteoblastogenesis in addition to Runx2 and COL1A2.

## STATEMENT

All procedures involving animals were approved by the Home Ofice (PIL 40/10118) and the University of Sheffield’s Animal Ethics Committee.
